# The patient voice: a survey of worries and anxieties during health system transition in HIV services in Vietnam

**DOI:** 10.1186/s12914-019-0221-7

**Published:** 2020-01-10

**Authors:** Shoko Matsumoto, Hoai Dung Thi Nguyen, Dung Thi Nguyen, Giang Van Tran, Junko Tanuma, Daisuke Mizushima, Kinh Van Nguyen, Shinichi Oka

**Affiliations:** 1 AIDS Clinical Center, National Center for Global Health and Medicine, 1-21-1, Toyama, Shinjuku, Tokyo, 162-8655 Japan; 2 National Hospital for Tropical Diseases, Hanoi, Vietnam; 3 Hanoi Medical University, Hanoi, Vietnam

**Keywords:** Social health insurance, HIV, Vietnam, Health system

## Abstract

**Background:**

Vietnam is shifting toward integrating HIV services into the public health system using social health insurance (SHI), and the HIV service delivery system is becoming decentralized. The study aim was to investigate current SHI coverage and patients’ perspectives on this transition.

**Methods:**

A survey of 1348 HIV-positive patients on antiretroviral therapy (aged ≥18 years) was conducted at an HIV outpatient clinic at a central-level hospital in Hanoi, Vietnam, in October and November 2018. Insurance coverage, reasons for not having a SHI card, perceived concerns about receiving HIV services in SHI-registered local health facilities, and willingness to continue regularly visiting the current hospital were self-reported. Logistic regression analyses were performed to analyze factors associated with not having a SHI card and having concerns about receiving HIV services in SHI-registered hospitals/clinics.

**Results:**

SHI coverage was 78.0%. The most frequently reported reason for not having a SHI card was that obtaining one was burdensome, followed by lack of information on how to obtain a card, and financial problems. Most patients (86.6%) had concerns about receiving HIV services at SHI-registered local health facilities, and disclosure of HIV status to neighbors and low quality of HIV services were the main concerns reported. Participants aged < 40 years old and unmarried were more likely to report lack of SHI cards, and women and those aged ≥40 years were more likely to have concerns. However, 91.4% of patients showed willingness to continue regular visits to the current hospital.

**Conclusions:**

Although SHI coverage has been rapidly improving among HIV patients, most participants had concerns about the current system transition in Vietnam. In response to their voiced concerns, strengthening the link between higher-level and lower-level facilities may help to ensure good quality HIV services at all levels while mitigating patients’ worries and anxieties.

## Background

Vietnam’s HIV epidemic has stabilized after peaking in the early 2000s. However, HIV still remains a public health threat in Vietnam, with an estimated 220,000 to 280,000 people living with HIV (PLHIV) and 11,000 new HIV infections in 2017 [1]. The HIV epidemic is concentrated in key populations, including people who inject drugs; 45% of new infections in 2013 occurred among men who shared needles when injecting drugs [2].

Over the last two decades, large international cooperation networks have supported antiretroviral therapy (ART) in Vietnam, including, but not limited to, the United States’ President’s Emergency Plan for AIDS Relief (PEPFAR) and the Global Fund to Fight AIDS, Tuberculosis and Malaria. However, as Vietnam’s economy has grown, these organizations have been phasing out their activities in Vietnam; the largest source of international aid, the PEPFAR program, will end its direct support for ART by the end of 2019.

To sustainably finance the maintenance and expansion of HIV care and treatment, Vietnam is now shifting toward integrating HIV services into the public health system using social health insurance (SHI). After 3 years of piloting voluntary health insurance schemes in some provinces, Vietnam introduced social health insurance to all provinces in 1992 to cover formal-sector workers and pensioners. With the goal of universal coverage, the Vietnamese government issued the first SHI law (Decree No. 63) in 2008, which expanded coverage to people experiencing poverty by fully subsidizing premiums for this group and informal-sector workers. To increase universal health coverage, the SHI law was revised in 2014 and SHI became mandatory for all citizens [3]. According to a report from the Vietnam Social Security (VSS), Vietnam has achieved a remarkable increase in SHI coverage; the number of health insurance card holders reached 83.5 million (88.5% of the total population) in 2019 [4]. As part of the transition from donor-based to SHI-based HIV service delivery, a Prime Minister’s Decision (No. 2188/QD-TTg) was issued in 2016 that introduced a new goal of 100% SHI coverage (hereafter referred to “universal SHI coverage”) for PLHIV by 2020. As a result, SHI coverage for PLHIV improved dramatically from 40% in 2014 [5] to more than 85% in 2018 [6].

The HIV finance transition includes the move from a centralized to a decentralized HIV service delivery system. As ART has been provided free of charge mainly owing to the support of international donors, many HIV patients are currently served by central urban hospitals and receive better quality of care. However, to be eligible for SHI coverage for HIV services, PLHIV must obtain a SHI card that can be used only in a SHI-registered health facility near their registered residence or in same-level facilities of a SHI-registered hospital/clinic in the same province. Although SHI coverage and decentralization improve access to HIV services at lower levels of care, they may pose new challenges for PLHIV who have been receiving ART free of charge at higher-level hospitals [7]. For example, many district- and commune-level HIV clinics have insufficient capacity and experience for HIV care and treatment, so that essential laboratory tests to monitor ART effectiveness, such as the viral load (VL) test, are often not available. Additionally, most PLHIV, except those experiencing poverty, will be expected to pay up to 20% of costs in copayments [5]. Furthermore, owing to the lack of privacy of health care, PLHIV may face difficulties keeping their HIV status confidential if they regularly visit local HIV clinics [8, 9].

The above-mentioned transition seems to be progressing rapidly owing to strong political commitments rather than patient-centered approaches [10]. To achieve universal health coverage and to ensure a decentralized service delivery system that meets the needs of PLHIV, it is important to listen carefully to patients’ voices to safeguard the continuation of effective, local HIV treatment, and to prevent patients from worrying about the new health system. Therefore, this study was designed to investigate the current SHI coverage situation and patients’ perspectives on the transition in the HIV service delivery system, and to provide possible solutions to reduce the negative impacts of the transition.

## Materials and methods

### Study design and study subjects

We conducted a self-administered questionnaire survey using a hospital-based cohort of PLHIV on ART (aged ≥18 years) in October and November 2018. This cohort, the so-called “Hanoi cohort,” was established in 2007 at the HIV outpatient clinic at the National Hospital for Tropical Diseases (NHTD), one of the largest central-level HIV clinics in Hanoi, Vietnam. At the time of the survey, the Hanoi cohort participants were still regularly receiving ART and other HIV services in NHTD, and they were invited to complete the survey during their regular consultations. Individuals who provided informed consent participated in the survey on the same day as their consultation.

To develop the questionnaire used in this survey, an expert panel was formed by HIV/AIDS specialists, including HIV clinicians, social workers, and a social epidemiologist. The panel developed the questionnaire and response options using information from previous relevant reports [10, 11] and experiences in day-to-day clinical practice.

### Measurements

#### SHI coverage

SHI coverage was evaluated by asking whether participants had a valid SHI card. The hospital/clinic level (i.e., national, provincial, district, or commune) was also recorded. Participants who did not have a SHI card were asked to provide reasons using the question “Why don’t you have a SHI card?” Possible responses were “I cannot afford it,” “It feels like a burden,” “I do not know how to get one,” and “Other.” Participants could select as many responses as were applicable. If they selected “Other,” they were able to provide reasons (although this was optional).

#### Concerns about receiving HIV services in SHI-registered hospitals/clinics

The presence of perceived concerns about receiving HIV services in SHI-registered hospitals/clinics was evaluated with the question “Do you have any concerns about receiving HIV services in your SHI-registered health facilities?”; possible responses were “yes” or “no.” Participants with perceived concerns were then asked about the nature of their concerns; possible responses were “Disclosure of HIV status to neighbors,” “Low quality of HIV services,” “Fear of not getting along with unfamiliar medical staffs,” and “Other.” Participants could select as many items as were applicable. If they selected “Other,” they were able to provide reasons (although this was optional).

#### Willingness to regularly visit the current hospital

Patients’ willingness to regularly visit their current hospital was evaluated with the question “Are you willing to continue visiting NHTD every 6 months for VL monitoring after being transferred to the SHI-registered local hospital/clinic?” Possible responses were “yes” or “no.” Participants who responded “yes” were further asked about their reasons, and possible responses were “I think that VL monitoring is beneficial for me,” “I want to keep in contact with NHTD,” “I want to meet staffs here,” and “Other.” Participants could select as many items as were applicable. If they selected “Other,” they were able to provide reasons (although this was optional).

#### Demographics and HIV-related factors

The following data on demographic and HIV-related factors were collected: sex, age, history of injection drug use (IDU), duration of receiving HIV services at NHTD, marital status, residence, educational attainment, employment, individual income, and disclosure of HIV status. Age was divided into two categories using the median value: < 40 years and ≥ 40 years. History of IDU was divided into two categories: IDU (ever used injection drugs) and non-IDU (never used injection drugs). Duration of receiving HIV services at NHTD was divided into the following categories using 25th and 75th percentile values: < 5 years, 5–9 years, and ≥ 10 years. Marital status was divided into two categories: married and not married (including divorced or widowed). Residence was divided into three categories: Hanoi, provinces around Hanoi, and other provinces. Provinces around Hanoi included eight provinces bordered by Hanoi and regarded as neighbor provinces. “Other provinces” referred to provinces that are a substantial traveling distance from Hanoi. Employment was categorized as not employed, employed, or retired. Individual income was divided into the following categories according to monthly income per capita in 2012, as reported by the General Statistics Office of Vietnam [12]: low (< 1,500,000 Vietnamese dong [VND]), middle (1,500,000–4,999,999 VND), and high (≥5,000,000 VND) (1 VND = 0.000043 USD). Disclosure of HIV status was evaluated dichotomously. Educational attainment was divided into three groups based on the fact that the primary school enrollment rate reached over 90% and the tertiary education enrollment rate reached 25 to 30% in 2018 [13]: low (never went to school, primary school, or junior high school), middle (high school), and high (vocational school/college or university).

### Statistical analysis

Baseline characteristics were summarized for all participants. We calculated SHI coverage, the proportion of participants who had concerns about receiving HIV services in SHI-registered hospitals/clinics, and the proportion of participants willing to regularly visit the current hospital, and then summarized the reasons for not having a SHI card, the nature of concerns, and reasons for the willingness to regularly attend the current hospital. Logistic regression analyses were then performed to analyze the factors associated with “not having a SHI card” and “having concerns about receiving HIV services in SHI-registered hospitals/clinics,” and crude and multivariate-adjusted odds ratios (OR) were calculated. In the multivariate analyses, we used variables that had *p-*values < 0.05 in the univariate analysis for adjustment. As a sensitivity analysis, we conducted multivariate analyses using a stepwise selection method for all variables (inclusion and exclusion criteria = 0.2 for each).

All analyses were performed using SAS 9.4 software (SAS Institute Inc., Cary, NC, USA). All tests were two-sided, with the significance level set at 5%. Missing data were excluded from the analyses.

## Results

### Study participants

Since October 2007, 1820 patients had registered for the Hanoi cohort, and 1348 were still enrolled and underwent follow-up in October and November 2018. All patients agreed to participate in the survey (response rate: 100%).

Table 1 shows the respondents’ characteristics. In all, 58.3% of participants were males. The median age (interquartile range) was 40 (36–45) years. Furthermore, 22.9% of participants had a history of IDU, and nearly 90% had been receiving HIV services at NHTD for 5 years or longer. Approximately one-third lived in provinces far from Hanoi (there was even one participant who lived in Hue, a city in the middle of Vietnam).

**Table 1:** Participant characteristics2

	n (%)
All	1348 (100.0)
Sex	
Male	786 (58.3)
Female	562 (41.7)
Age	
Median (IQR)	40 (36, 45)
< 40 years old	612 (45.4)
≥ 40 years old	736 (54.6)
History of IDU	
No	1040 (77.2)
Yes	308 (22.9)
Duration of receiving HIV services at NHTD	
< 5 years	154 (11.4)
5–9 years	910 (67.5)
≥ 10 years	284 (21.2)
Marital status	
Married	974 (72.3)
Not married	139 (10.3)
N/A	235 (17.4)
Residence	
Hanoi	607 (45.0)
Provinces around Hanoi	259 (19.2)
Other provinces	482 (35.8)
Educational attainment^a^	
Low	291 (21.6)
Middle	345 (25.6)
High	477 (35.4)
N/A	235 (17.4)
Employment	
Not employed	234 (17.4)
Employed	821 (60.9)
Retired	54 (4.0)
N/A	239 (17.7)
Individual income^b^	
Low	264 (19.6)
Middle	527 (39.1)
High	259 (19.2)
N/A	298 (22.1)
Disclosure of HIV status	
No	57 (4.2)
Yes	1056 (78.3)
N/A	235 (17.4)

*N/A* missing value, *IDU* injection drug use, *IQR* interquartile range, *NHTD* National Hospital for Tropical Diseases

^a^Educational attainment: low: never went to school, primary school, or junior high school; middle: high school; high: vocational school/college or university

^b^Individual income: low: < 1,500,000 VND; middle: 1,500,000–4,999,999 VND; high: ≥5,000,000 VND

### SHI coverage and reasons for not having a SHI card

**Fig. 1:**
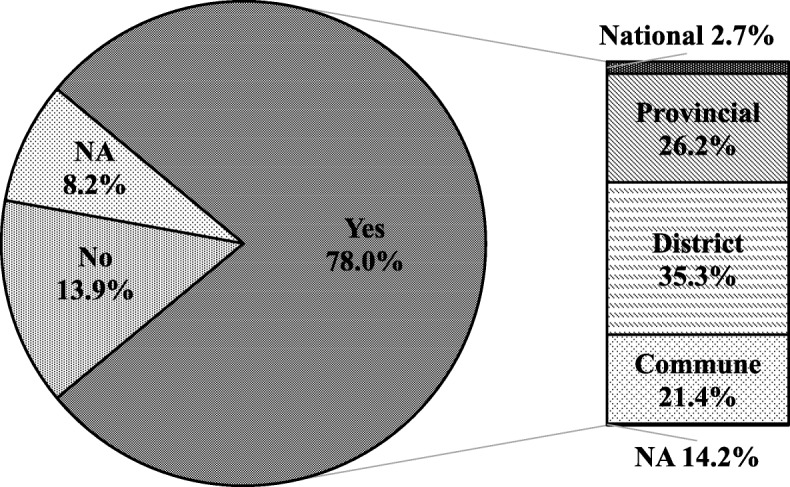
SHI coverage and level of SHI-registered facilities. NA: data are not available. SHI: social health insurance

**Fig. 2:**
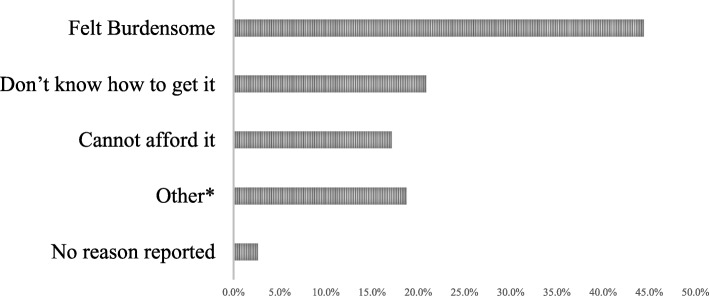
Reasons for not having a SHI card. *Other (*n* = 35) includes “fear of HIV disclosure” (7), “feeling no necessity” (4), “just changed jobs” (3), “no time” (2), “forgot” (1), and “NA” (18). SHI: social health insurance

In total, 1051 (78.0%) participants had a SHI card; of these, 35.3% were registered at district-level hospitals/clinics and 21.4% at commune-level hospitals/clinics (Fig. 1). Of the 187 participants who did not have a SHI card, the most frequently reported reason was that obtaining a card “felt burdensome” (44.4%), followed by “don’t know how to get it” (20.9%) and “cannot afford it” (17.1%). Some other reasons reported were “fear of revealing HIV status” and “don’t feel that it’s necessary” (Fig. 2).

### Concerns about receiving HIV services in SHI-registered hospitals/clinics

**Fig. 3:**
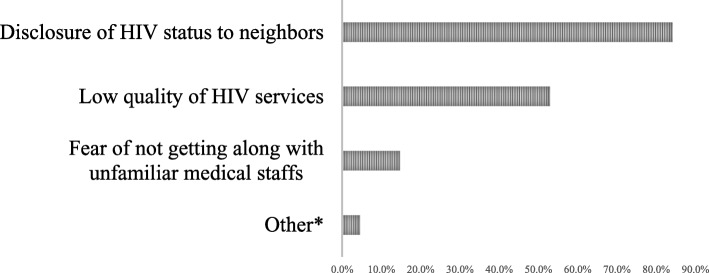
Concerns about receiving HIV services in SHI-registered hospitals/clinics. *Other (*n* = 54) includes “not wishing to change hospital” (33), “influence on work or family” (17), and “inconvenience” (4). SHI: social health insurance

In total, 1167 (86.6%) participants had concerns about receiving HIV services in SHI-registered hospitals/clinics. Of these, 84.2% were concerned about disclosure of their HIV status to neighbors and 53.0% were concerned about the low quality of HIV services. Fear of not getting along with unfamiliar medical staffs was less frequently reported (14.8%). Some other concerns reported were “not wishing to change hospital” and “influence on work or family” (Fig. 3).

### Willingness to regularly visit the current hospital

**Fig. 4:**
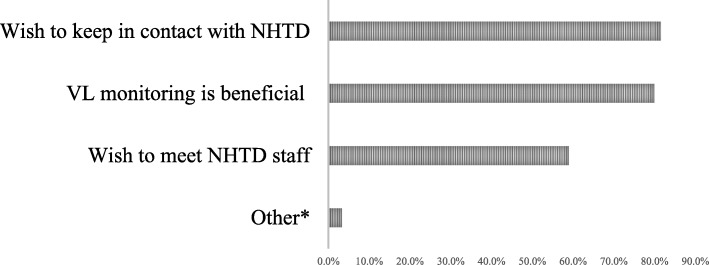
Reasons for being willing to regularly visit NHTD. *Other (*n* = 41) includes “not wishing to change hospital” (16), “satisfied with quality of service at NHTD” (15), “convenience” (5), “fear of HIV disclosure” (3), and other (2). NHTD: National Hospital for Tropical Diseases; SHI: social health insurance; VL: viral load

There were 1232 (91.4%) participants who were willing to continue visiting NHTD after being transferred to SHI-registered local hospitals/clinics. The most frequently reported reason for their willingness was “I want to keep in contact with NHTD” (81.5%) followed by “I think that VL monitoring is beneficial for me” (79.9%) and “I want to meet staffs here” (58.9%) (Fig. 4).

### Factors associated with not having a SHI card and having concerns about receiving HIV services in SHI-registered hospitals/clinics

Table 2 shows ORs and 95% confidence intervals (95% CI) for the univariate and multivariate logistic regression models with not having a SHI card as the outcome variable.

**Table 2:** ORs and 95% CIs for not having a SHI card: regression results

	Univariate model		Multivariate model^a^ (*n* = 1020)	
	OR (95% CI)	*p*-value	OR (95% CI)	*p*-value
Sex				
Male	1.00	0.21		
Female	0.82 (0.59–1.12)			
Age				
< 40 years old	1.48 (1.09–2.03)	0.01	1.46 (1.02–2.08)^d^	0.04
≥ 40 years old	1.00		1.00	
History of IDU				
No	1.35 (0.90–2.01)	0.14		
Yes	1.00			
Duration of receiving HIV services at NHTD				
< 5 years	1.00	0.20		
5–9 years	0.92 (0.57–1.48)			
≥ 10 years	0.64 (0.36–1.14)			
Marital status				
Married	1.00	0.02	1.00	0.04
Not married	1.74 (1.09–2.77)		1.64 (1.02–2.62)^d^	
Residence				
Hanoi	1.00	0.31		
Provinces around Hanoi	0.99 (0.66–1.48)			
Other provinces	0.76 (0.53–1.09)			
Educational attainment^b^				
Low	0.70 (0.44–1.12)	0.23		
Middle	1.05 (0.71–1.57)			
High	1.00			
Employment				
Not employed	1.13 (0.74–1.72)	0.32		
Employed	1.00			
Retired	0.49 (0.17–1.39)			
Individual income^c^				
Low	1.12 (0.66–1.90)	0.59		
Middle	1.26 (0.80–1.97)			
High	1.00			
Disclosure of HIV status				
No	1.91 (1.00–3.64)	0.05		
Yes	1.00			

*OR* odds ratio, *95% CI* 95% confidence interval, *IDU* injection drug use, *NHTD* National Hospital for Tropical Diseases, *SHI* social health insurance

^a^Variables with *p*-values < 0.05 in the univariate analysis were used for adjustment

^b^Educational attainment: low: never went to school, primary school, or junior high school; middle: high school; high: vocational school/college or university

^c^Individual income: low: < 1,500,000 VND; middle: 1,500,000–4,999,999 VND; high: ≥5,000,000 VND

^d^Age (< 40 years old) (OR = 1.47, 95% CI: 1.02–2.12 vs. ≥40 years old) and not being married (OR = 1.66, 95% CI: 1.01–2.72 vs. being married) were associated with not having a SHI card (*p*-values of 0.040 and 0.045, respectively) in the multivariate model using stepwise selection method for all variables (inclusion and exclusion criteria = 0.2 for each)

In the univariate model, age (< 40 years old) (OR = 1.48, 95% CI: 1.09–2.03 vs. ≥40 years old) and not being married (OR = 1.74, 95% CI: 1.09–2.77 vs. being married) were significantly associated with not having a SHI card. In the multivariate model, both age (< 40 years old) (OR = 1.46, 95% CI: 1.02–2.08 vs. ≥40 years old) and not being married (OR = 1.64, 95% CI: 1.02–2.62 vs. being married) remained significant, which was consistent with the results of the multivariate model using the stepwise selection method.

Table 3 shows ORs and 95% CIs for the univariate and multivariate logistic regression models with having concerns about receiving HIV services in SHI-registered hospitals/clinics as the outcome variable. In the univariate model, being female (OR = 2.05, 95% CI: 1.45–2.89 vs. male), age (≥40 years old) (OR = 1.39, 95% CI: 1.02–1.90 vs. < 40 years old), non-IDU (OR = 1.68, 95% CI: 1.19–2.37 vs. IDU), and being married (OR = 1.88, 95% CI: 1.19–2.95 vs. not being married) were significantly associated with having concerns about receiving HIV services. Of these, female sex (OR = 2.02, 95% CI: 1.33–3.08 vs. male) and age (≥40 years old) (OR = 1.46, 95% CI: 1.02–2.08 vs. < 40 years old) remained significant in the multivariate model, which is consistent with the results of the sensitivity analysis using the stepwise selection method.

**Table 3:** ORs and 95% CIs for having concerns about HIV services in SHI-registered hospitals/clinics: regression results

	Univariate model		Multivariate model^a^ (*n* = 1113)	
	OR (95% CI)	*p*-value	OR (95% CI)	*p*-value
Sex				
Male	1.00	< 0.001	1.00	< 0.01
Female	2.05 (1.45–2.89)		2.02 (1.33–3.08)^d^	
Age				
< 40 years old	1.00	0.04	1.00	0.04
≥ 40 years old	1.39 (1.02–1.90)		1.46 (1.02–2.08)^d^	
History of IDU				
No	1.68 (1.19–2.37)	< 0.01	1.05 (0.68–1.61)	0.84
Yes	1.00		1.00	
Duration of receiving HIV services at NHTD				
< 5 years	1.00	0.27		
5–9 years	1.22 (0.76–1.94)			
≥ 10 years	1.58 (0.90–2.79)			
Marital status				
Married	1.88 (1.19–2.95)	0.01	1.56 (0.98–2.49)	0.06
Not married	1.00		1.00	
Residence				
Hanoi	1.00	0.60		
Provinces around Hanoi	1.07 (0.70–1.63)			
Other provinces	1.20 (0.84–1.71)			
Educational attainment^b^				
Low	0.72 (0.47–1.10)	0.22		
Middle	0.74 (0.49–1.11)			
High	1.00			
Employment				
Not employed	0.94 (0.62–1.43)	0.84		
Employed	1.00			
Retired	1.24 (0.52–2.96)			
Individual income^c^				
Low	0.80 (0.47–1.35)	0.70		
Middle	0.87 (0.55–1.38)			
High	1.00			
Disclosure of HIV status				
No	1.00	0.35		
Yes	0.71 (0.35–1.44)			
Level of SHI-registered hospital/clinic				
National	1.00	0.52		
Provincial	1.71 (0.67–4.37)			
District	1.34 (0.53–3.34)			
Commune	1.21 (0.47–3.08)			
Unknown	0.60 (0.05–6.79)			

*OR* odds ratio, *95% CI* 95% confidence interval, *IDU* injection drug use, *NHTD* National Hospital for Tropical Diseases, *SHI* social health insurance

^a^Variables with *p*-values < 0.05 in the univariate analysis were used for adjustment

^b^Educational attainment: low: never went to school, primary school, or junior high school; middle: high school; high: vocational school/college or university

^c^Individual income: low: < 1,500,000 VND; middle: 1,500,000–4,999,999 VND; high: ≥5,000,000 VND

^d^Being female (OR = 2.46, 95% CI: 1.56–3.88 vs. male) and age (≥40 years old) (OR = 1.58, 95% CI: 1.04–2.40 vs. < 40 years old) were associated with having concerns about receiving HIV services in SHI-registered hospitals/clinics (*p*-values < 0.001 and 0.03, respectively) in the multivariate model using stepwise selection method for all variables (inclusion and exclusion criteria = 0.2 for each)

## Discussion

In this study, we evaluated patients’ perspectives on the current transition of the HIV service delivery system in Vietnam. The SHI coverage was 78.0%; over half the participants were SHI-registered at district- or commune-level clinics. Most participants had concerns about receiving HIV services in SHI-registered hospitals/clinics; the most frequently reported concerns were disclosure of HIV status to neighbors and low quality of HIV services. Over 90% of participants were willing to continue regularly visiting NHTD after being transferred to SHI-registered local clinics.

### Toward universal SHI coverage

The transition of HIV finance is not an issue only in Vietnam. As international funding decreases, many low- and middle-income countries are now making efforts to increase domestic resources to fill the gap [14, 15]. In 2018, approximately 56% of the total resources for HIV in low- and middle-income countries were from domestic sources [16]. Although challenging for those countries, a shift toward domestic funding has advantages in terms of ownership, accountability, and sustainability of the national HIV response [17].

At NHTD, one of the largest HIV clinics in Hanoi, the SHI coverage was 78%, indicating that SHI enrollment has been rapidly progressing (40% in 2014 [5]), but that universal SHI coverage has not been reached. Although previous reports have highlighted financial burden as the main barrier to SHI access [11, 18], in this study, the most frequently reported reasons for not having a SHI card were that obtaining one was burdensome and there was a lack of information about accessing SHI, rather than financial problems. In addition, two finance-related variables (individual income and employment) were not associated with lack of a SHI card in the logistic regression analyses. Various recent policies and schemes introduced by central and local government (e.g., subsidies for premiums for people living in poverty and near-poverty, allocation of local budgets for purchasing SHI cards) might have successfully contributed to the expansion of SHI. This interpretation should be tested in other settings, as our study participants at NHTD may be receiving better financial support from local government and other authorities; such support may vary between provinces depending on their financial resources and commitment to HIV prevention and control.

Feeling that it was burdensome to obtain a SHI card and lacking information on how to obtain a SHI card were the most frequently reported reasons for not having one, and younger age (< 40 years old) and being unmarried were identified as possible risk factors. A burdensome feeling could arise for various reasons, including complicated administrative procedures for SHI registration, long waiting times, or feeling that a card is not necessary [11]. As SHI cards are issued based on residential information, disclosure of HIV status to others at the time of obtaining a card may be an additional reason for not having one. Alternatively, the reported lack of information about obtaining SHI cards may reflect patient lack of interest in SHI, as well as problems related to availability and accessibility of information. In particular, it may be difficult for younger people to understand the benefits of SHI, and unmarried persons might be less motivated to enroll into SHI. As previously suggested, providing adequate information about SHI could encourage the enrollment of PLHIV in SHI [7, 11, 19]. In a previous study, we found that family is often the only and the strongest supporter of PLHIV [20]. Raising awareness of the necessity and benefits of SHI, especially among younger and unmarried individuals, and providing support tailored to individual needs (e.g., the most convenient way to register for SHI) for both patients and family could enhance the understanding of SHI enrollment and patients’ willingness to join SHI and help to achieve universal SHI coverage.

### Patients’ worries and anxieties during decentralization

#### HIV stigma in the community

Participants’ biggest concern about the decentralization of the HIV service delivery system was disclosure of HIV status to neighbors. In Vietnam, the HIV epidemic is concentrated among high-risk groups (e.g., IDU and sex workers) and many PLHIV live with substantial HIV stigma [21–23]. Over half of the study participants live outside Hanoi, and seeking HIV services far from home is a way of keeping their HIV status confidential and avoiding the negative consequences of disclosure (e.g., effects on work and family). It is well known that HIV-related stigma is the main barrier to accessing HIV services [24, 25]. Therefore, the Vietnamese Ministry of Health issued a stigma reduction guideline in health care settings in 2017 and encouraged HIV clinics to develop an action plan for stigma reduction. However, PLHIV experience stigma from their community more frequently than from health care providers [9].

Over the past decade, there has been remarkable progress in research on interventions for stigma reduction, and an increase in the number and quality of intervention strategies [26]. Many research interventions have focused not only on PLHIV and health care workers, but also on local government, non-governmental organizations, community leaders or members, students, family, and women [26, 27]. Some researchers have suggested that interventions that combine multiple strategies (e.g., information-based approaches, skills building, counseling/support, and contact with affected groups) to address multiple stigma domains (e.g., drivers, facilitators, intersecting stigmas, and manifestations of stigma) may be more effective [26–28]. During the HIV service delivery transition, policymakers in Vietnam need to learn lessons from other countries and focus on expanding stigma reduction activities in various fields, to ensure that PLHIV can receive local HIV services without experiencing stigma.

#### Low quality of health services at lower-level health facilities

Notably, over half of study participants were concerned about the low quality of services in SHI-registered hospitals/clinics. Historically, HIV services in Vietnam have been provided through the preventive medicine system. To decentralize the HIV service delivery system, the Ministry of Health has encouraged prevention-focused facilities to sign contracts with the VSS to allow provision of and reimbursement for HIV/AIDS curative services through the SHI fund [5]. However, HIV patients do not yet trust the quality of health services at the new local facilities that have signed contracts with the VSS. Like our study participants, many PLHIV have been receiving ART at urban hospitals for a long time, and their VL has been successfully controlled [29]. In this context, it is easy to understand that PLHIV worry about being transferred to lower-level facilities just for political reasons, rather than to meet their own needs.

In the present study, many participants showed a willingness to regularly visit NHTD after being transferred to SHI-registered hospitals/clinics. This suggests that strengthening links between higher-level and lower-level facilities to continuously transfer knowledge and techniques for HIV treatment and care is a possible way to ensure the good quality of HIV services at all levels and to mitigate patients’ worries and anxieties. Although NHTD and other large HIV clinics have been organizing HIV training courses for medical doctors who have just started providing HIV treatment, these are mainly classroom lectures based on project-based grants from international organizations. The Vietnamese Ministry of Health must show stronger leadership to establish an effective hospital network to promote technical assistance in more systematic and practical ways. This would certainly help to achieve 90–90–90 targets in Vietnam.

#### Factors associated with concerns about decentralization

We found that female and older (≥40 years old) participants were more likely to report concerns about the decentralization of HIV services. Considering the influence of gender roles on childbearing, women are more likely to be concerned about influences on their children [30]. Indeed, some participants reported that “It will influence my child’s school life” and “It will stress my children.” Alternatively, the association between older age and having concerns about decentralization might be partly explained by the double stigma of HIV and ageism (i.e., patients are recognized as being too old to have HIV) and higher demands on the quality of health services to address multiple health complications [31]. Considering that women and older people are more likely to develop psychiatric disorders such as depression [32, 33] and anxiety [34, 35], which may be triggered by loneliness or social isolation [36, 37], more attention should be paid to their unique needs to prevent mental illness in these groups.

### Strengths and limitations

A strength of the present study is the uniqueness of the data, which reflect patients’ voices about the current HIV financial and service transition obtained from one of the largest HIV patient cohorts in Vietnam, with a response rate of 100%. These findings could contribute to the development of strategies based on patients’ perspectives to successfully implement the decentralized HIV service delivery using the SHI fund. However, a few limitations should be acknowledged. First, as this was a single-center study, the participants who were receiving HIV services at a central urban hospital may not be representative of Vietnam’s entire HIV population. Second, we used a cross-sectional design, which limits the extent to which causal inferences can be drawn for the associations identified. Third, as the purpose of the survey was to urgently assess the needs of HIV patients, the response options that we generated may have been too narrow to fully reflect their opinions. However, these responses were selected based on previous reports and experts’ opinions about HIV in Vietnam.

## Conclusions

Although SHI coverage has been rapidly progressing, most patients are concerned about disclosure of their HIV status and the quality of services at lower-level facilities, and are willing to maintain contact with their current hospital. In response to their voiced concerns, the establishment of a hospital network to strengthen the links between higher-level and lower-level facilities could facilitate good quality HIV services at all levels, mitigate patients’ worries and anxieties, and help to achieve the 90–90–90 targets in Vietnam.

## Data Availability

Not applicable.

## Abbreviations

95% CI: 95% confidence interval
ART: Antiretroviral therapy
IDU: Injection drug use
NHTD: National Hospital for Tropical Diseases
OR: Odds ratio
PEPFAR: The United States’ President’s Emergency Plan for AIDS Relief
PLHIV: People living with HIV
SHI: Social health insurance
VL: Viral load
VSS: Vietnam Social Security
